# Representation effects in the centipede game

**DOI:** 10.1371/journal.pone.0204422

**Published:** 2018-10-04

**Authors:** Paolo Crosetto, Marco Mantovani

**Affiliations:** 1 Univ. Grenoble Alpes, INRA, CNRS, Grenoble INP, GAEL, 38000 Grenoble, France; 2 DEMS, Università degli studi di Milano Bicocca, P.za dell’Ateneo Nuovo 1, 20126 Milan, Italy; Universidad Loyola Andalucia, SPAIN

## Abstract

We explore the effects on strategic behavior of alternative representations of a centipede game that differ in terms of complexity. In a laboratory experiment, we manipulate the way in which payoffs are presented to subjects in two different ways. In both cases, information is made less accessible relative to the standard representation of the game. Results show that these manipulations shift the distribution of take nodes further away from the equilibrium prediction. The evidence is consistent with the view that failures of game-form recognition and the resulting limits to strategic reasoning are crucial for explaining non-equilibrium behavior in the centipede game.

## Introduction

The effects on observed behavior of apparently superficial changes in presentation are generally referred to as framing effects. Their existence suggests that the game agents play is hardly ever identical to the canonical representation assumed by theory (see, in general, [1]; for application to games, see [2, 3]; for recent evidence on decision processes in games, see [4, 5]).

By manipulating the presentation of information about payoffs, we perform two manipulations on the way a simple centipede game is presented to subjects to gather insights on the commonly observed patterns of behavior in this game. Assuming that the manipulated presentations are more complex than the standard format, we interpret our results as isolating the effects on behavior of (marginal) increases in complexity in a simple sequential game.

The centipede game [6] is a two-player game in which players alternate choosing whether to end the game (“take”) or to pass to the other player (“pass”). The payoff from taking in the current decision node is greater than that received in case the other player takes in the next one, but less than the payoff he could potentially earn if the other player were to pass as well. The player making the final choice is paid more from taking than from passing, and would therefore be expected to take. Iterating this argument, backward induction leads to the unique subgame perfect equilibrium: the game is stopped at the first decision node. The experimental evidence [7–10] shows that players fail to comply with this extreme unraveling prediction.

Explanations of deviations from equilibrium are diverse. Some rely on non-standard preferences, or beliefs about preferences [7, 11, 12]. Others on bounded strategic thinking, including departures from common knowledge of rationality, incorrect beliefs, imperfect best responses and ambiguity [13–16]. [17, 18] argue that the typical empirical patterns that are observed in experimental data can be understood within a learning model, where initial deviations are due to a failure to appreciate the strategic environment.

In order to appreciate the strategic environment of the centipede game, players need to process information about payoffs that are distant from the current decision node. In our experiment, we manipulate how accessible this information is. We make information less accessible to throw sand in the gearbox of strategic thinking. As a consequence, subjects are expected to choose to end the game later, as it takes longer to recognize the strategic structure of the game. We then test whether our manipulations shift behavior away from the equilibrium solution.

In our baseline treatment (*Tree*), players are shown the standard extensive-form game, displaying the payoffs that follow each terminal history. In treatment *Formula*, players are informed only about the (simple) rule that governs payoffs throughout the game, and have to compute the final payoffs at future terminal histories. In treatment *Decomposed*, payoffs are decomposed in stage payoffs: subjects are informed of the change in payoffs that follows each decision to pass. This manipulation is similar to the ones in [19] and [20] on the decomposed Prisoner Dilemma. To compute the final payoffs, they need to sum up the stage payoffs to each terminal history. The rules of the game and their description, as well as final payoffs and all other details of the design, are identical across treatments. In particular, the same information is available to players in each treatment, although it is presented in different ways.

Results show subjects choose to take significantly later in treatments *Formula* and *Decomposed*, and the difference with the baseline persists over repetitions. Treatment *Decomposed* presents the payoffs with a *give-and-take* frame. One may think this presentation could elicit reciprocal behavior, as it highlights the similarity of the centipede with a repeated trust game. However, regression analysis shows that treatment effects are not mediated by preferences. Taken together, these results support our interpretation that treatment effects can be attributed directly to the reduction in accessibility of information.

The paper is organized as follows. In the next section, we describe the experimental design and present our main hypothesis. The actual implementation of the design in the lab is detailed in Section “Experimental procedure”. Section “Results” describes the results, and Section “Discussion and conclusion” concludes.

## Experimental design

**Fig 1:**
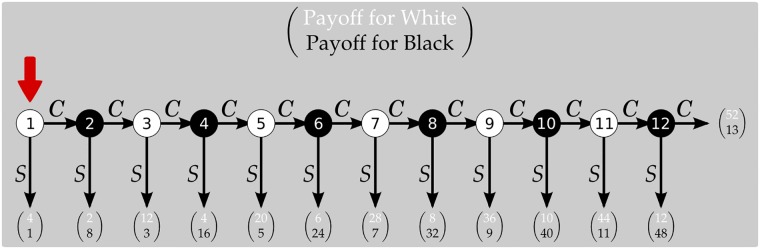
The representation of the game in the *Tree* condition, payoffs in euro.

We implement a twelve-legged centipede (see Fig 1). Actions are labeled “Stop” and “Continue”. Terminal histories are ordered and assigned a number *t* ∈ {1, …, 13} (1: Stop at first node; …; 13: Always continue). The aggregate payoff of the two players at any terminal history is worth 5 times the corresponding terminal history number; the player choosing “Stop” gathers $\frac{4}{5}$ of the cake—i.e., 4*t*—while the opponent gathers the remaining $\frac{1}{5}$—i.e., *t*.

We choose an arithmetic progression rather than the more common, geometric one [7] because the linear increase over nodes makes the underlying formula easy to convey also to subjects with potentially low numeracy skills. On top of being difficult to convey, a similar transformation with a different progression of payoffs would imply a different manipulation of strategic complexity. As we discuss in the conclusion our results do not obviously extend to all possible manipulations of complexity.

**Fig 2:**
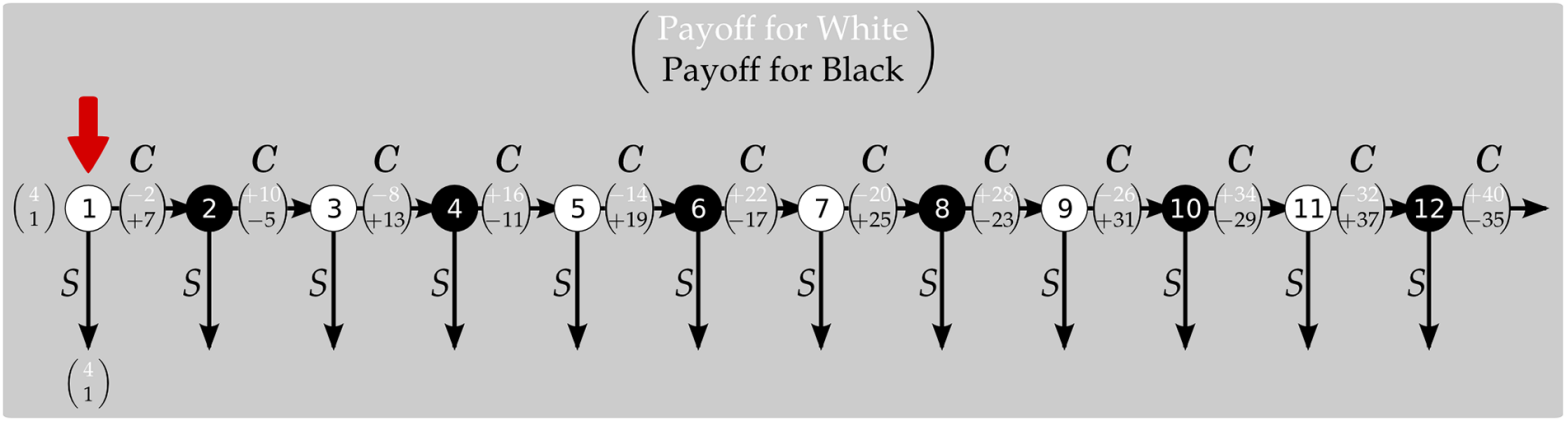
The representation of the game in the *Decomposed* condition, payoffs in euro.

We implement three different ways of conveying the payoff information:

**Tree**: subjects are shown the extensive-form representation of the game, as shown in Fig 1. That is, the game tree and the payoffs accruing to each player at every terminal history.
**Formula**: subjects are told the rule that determines the payoffs of the game, explained both in words and through the corresponding algebra. The exact formulation, as reported in the experimental instructions is: “When a player chooses STOP at round = r, the value for him is 4 times the current round, that is: *V*_*STOP*_ = 4 ⋅ *r*. The value for the other player is 1 times the current round, that is *V*_*OTHER*_ = 1 ⋅ *r*”.
**Decomposed**: subjects are shown a representation of the game providing them with stage payoffs, rather than the terminal ones, as shown in Fig 2. That is, they are given the game tree and the variation in payoffs that descends from any decision to pass.

The images in Figs 1 and 2 were both given to the subjects in a printed version as part of their instructions, and presented on screen at every decision node (including decision nodes where the player is inactive). In the screen version, the red arrow would move to indicate the current decision node; moreover, all past decision nodes would gray out.

Under all treatment conditions, players are given the same information, the only difference being how accessible this information is. In conditions *Formula* and *Decomposed*, players have to compute payoffs for future terminal histories on their own. In our context, this step is minimally demanding: it requires the computation of the four-times table or of simple integer sums, respectively.

One class of explanations for departures from equilibrium in the centipede game is that they are due to failures of strategic reasoning, or beliefs of failures of strategic reasoning on the part of the opponent. For instance, the equilibrium solution can be found by backward induction. Even if players fail to appreciate the strategic environment at the beginning of the game, sooner or later, one player discovers the solution for the remaining subgame, or believes his opponent is about to do so, and chooses ‘Stop’. We hypothesize that making information less accessible delays the moment this happens, as players find it harder to solve any given subgame, or believe this will hold for their opponent. This rightward shift of take nodes is not only consistent with limits to backward reasoning, but also with other types of failures of strategic reasoning, such as Level-*k* models or Quantal Response Equilibrium. In the former, making information less accessible may lower the level of players; in the latter, it may make errors more likely. In both cases this would imply ending the game later.

**Hypothesis**: In conditions *Formula* and *Decomposed*, subjects choose “Stop” later than in condition *Tree*.

## Experimental procedure

The computerized experiment was run in Jena, Germany, in June 2012, involving 210 subjects distributed over 8 experimental sessions.

Recruitment was done with ORSEE [21] from the database of experimental subjects of the Max Planck Institute of Economics in Jena. Subjects sign an informed consent agreement when entering the database; they then voluntarily sign up for experiments, and are randomly allocated to sessions. Most of the subjects were students at the university of Jena (12% economists). Average age was 23.5 years (st.dev: 3.43, min: 18; max: 37), and 63% of the sample was composed by women. Though the sample was not representative of the general population at large, it is fairly representative of the population of Jena, a very young university town.

Seventy-four subjects took part in three baseline *Tree* sessions. Seventy-two subjects participated in three *Formula* sessions. Sixty-four in two *Decomposed* sessions. The experiment lasted about 1 hour, and average payoff across all sessions and conditions amounted to 11.8 euro, including a 2.5 euro show-up fee.

All sessions followed an identical procedure. After subjects were allowed into the lab, instructions were read aloud and extra time was given to the subjects to go through them on their own. Then all subjects had to correctly answer a set of control questions before being allowed to proceed.

After all subjects had cleared the control questions, the experiment started. Subjects were randomly assigned to their roles (“White” or “Black”), randomly matched, and proceeded to play the game. The same game was repeated 12 times, in fixed roles. We adopted a *perfect stranger* matching design, implemented using the turnpike protocol. This matching is such that subjects never play with the same partner twice, and that their partners never interact with one another. Since we inform subjects about this feature of the design, this reduces significantly the likelihood of observing contagion effects. The pairs were allowed to proceed each at their own pace within the 12 decision nodes of the game, but had to wait for the others between repetitions.

After completing the 12^*th*^ repetition, subjects were paid according to the results of a randomly drawn repetition, and were asked to fill in a questionnaire. We gathered qualitative information on their expectations about the game, the strategy they adopted, and their beliefs on opponents’ behavior. Moreover, we elicited self-reported quantitative measures of trust and risk aversion (using the SOEP German Panel trust and risk questions. For the risk question, see [22]) and of the perceived complexity of the task.

The experiment was conducted in German. The English translation of the experimental instructions is available in Appendix 1. The original German instructions, along with the experimental software (developed using zTree, [23]) are available upon request. The raw, anonymized data are available at the OSF repository https://osf.io/srqpc/.

## Results

Consistent with the literature, subjects did not comply with subgame perfect equilibrium predictions. The average endnode was 3.52 in the baseline, 4.18 and 4.17 in conditions *Formula* and *Decomposed*, respectively. Thus, in both *Formula* and *Decomposed* subjects choose to stop about $\frac{2}{3}$ of an endnode *later* than in the baseline.

**Fig 3:**
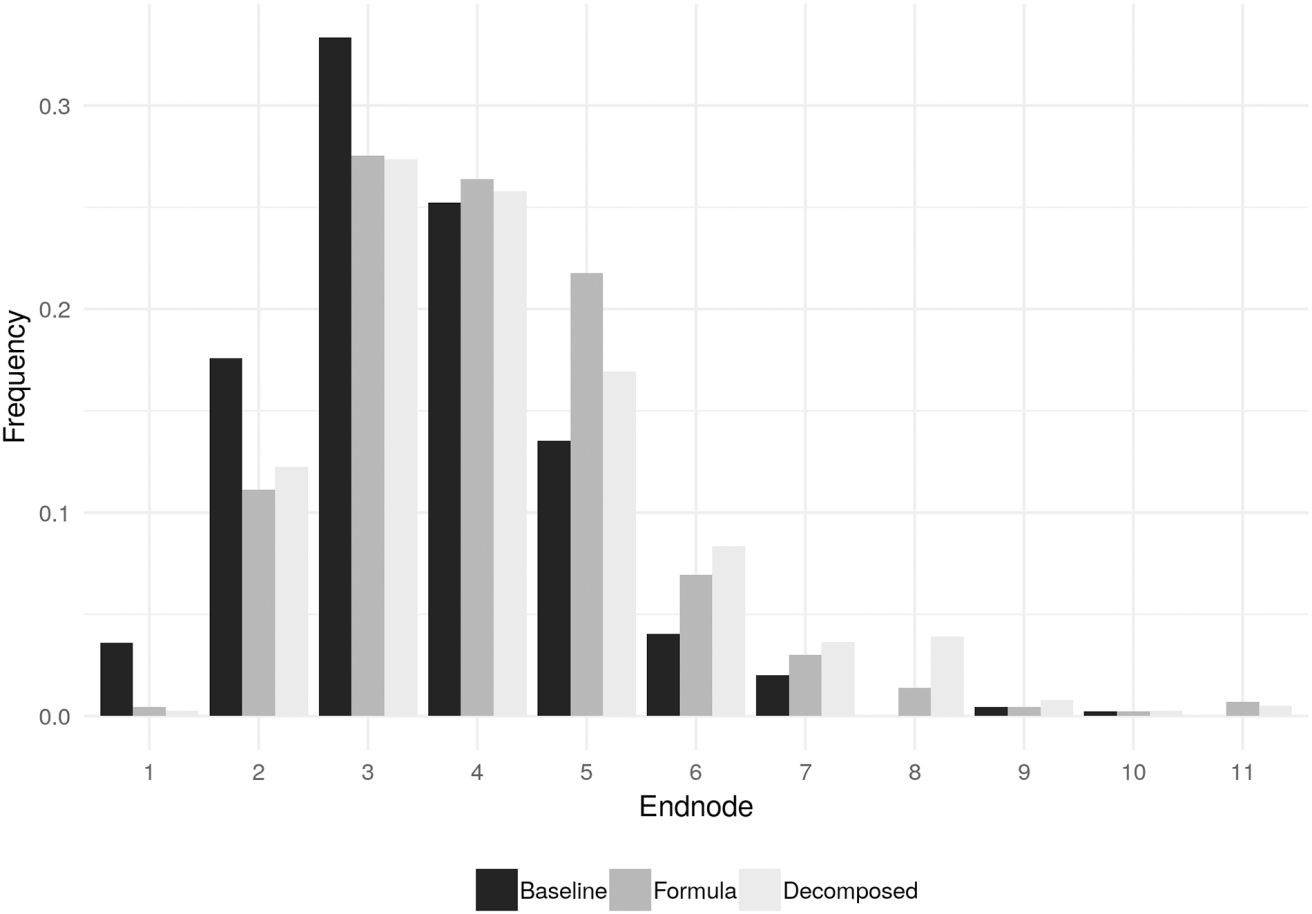
Distribution of endnodes in each treatment. Notes: the Figure shows, separately for each treatment, the distribution of the endnodes over the twelve repetitions of the game.

Fig 3 shows the distribution of endnodes in each of the three treatments. The distribution of endnodes in *Decomposed* and *Formula* are shifted to the right relative to that in *Tree*: the probability that a random game would reach at least node *t* is higher in *Formula* and *Decomposed* for all *t*. A Kruskal-Wallis test rejects the null of equal median across the three treatments (*z* = 39.63, *p*-value < 0.01). Following a similar non-parametric approach for pairwise comparisons, a Wilcoxon rank-sum tests rejects the null of an equal median for the comparison of the *Tree* condition with each of the *Formula* and *Decomposed* conditions, while it cannot reject it for the comparison of the latter two. Details are reported in Table 1. All non-parametric tests are based on one independent observation per pair. We are aware that, despite the use of the perfect stranger matching protocol, observations within a session are not independent as session effects could persist [24]. We hence run regressions directly addressing non-independence by clustering standard errors at the session level. The corresponding results, reported in Table 2, column (1), come to the same conclusion obtained with the non-parametric tests.

**Table 1:** Non-parametric tests of hypothesis.

	*Tree* vs *Formula* Wilcoxon	*Tree* vs *Decomposed* Wilcoxon	*Formula* vs *Decomposed* Wilcoxon	Threefold comparison Kruskal-Wallis
z (*p*-value)	**-5.625** **(.000)**	**-5.497** **(.000)**	-.259 (.795)	**39.673** **(.000)**

*Notes*: the first three columns of the table report the Wilcoxon rank-sum test, and corresponding p-value, on the pairwise difference between treatments. A negative and significant statistic means that the median endnode is higher for the second term of the comparison. The last column reports the Kruskal-Wallis test, and corresponding p-value, for the comparison of the medians among the three treatments. A significant statistic means that medians in the three treatments do not come from the same population. All tests are based on one independent observation for each pair of matched subjects—i.e. 444, 432, 384 ind. obs. in treatment *Tree*, *Formula*, and *Decomposed*, respectively. Bold indicates significance at the.01 level.

**Table 2:** OLS regressions by group.

	Dep. var.: endstage				
	(1)	(2)	(3)	(4)	(5)
*Formula*	0.556** (0.163)	0.773** (0.223)	0.521*** (0.129)	0.484* (0.321)	0.552** (0.209)
*Decomposed*	0.646** (0.273)	0.724* (0.486)	0.697** (0.282)	0.864** (0.304)	0.718** (0.300)
repetition		-0.210*** (0.0138)	-0.226*** (0.0153)	-0.226*** (0.0148)	-0.225*** (0.0149)
*Formula* × repetition		-0.0334 (0.0237)			
*Decomposed* × repetition		-0.0119 (0.0394)			
min(*trust*)			0.0786 (0.0858)		0.142 (0.190)
min(*risk*)			0.118*** (0.0275)	0.140** (0.0535)	
min(*complexity*)			-0.00470 (0.0551)		
min(*errors*)			-0.0222 (0.204)		
min(*timequiz*)			-0.000902 (0.000832)		
*Formula* × min(*trust*)				-0.00247 (0.0758)	
*Decomposed* × min(*trust*)				-0.0721 (0.0581)	
*Formula* × min(*risk*)					0.112 (0.228)
*Decomposed* × min(*risk*)					-0.205 (0.192)
Intercept	3.520*** (0.121)	4.885*** (0.0315)	4.648*** (0.183)	4.528*** (0.236)	4.940*** (0.160)
*N*	1260	1260	1260	1260	1260
R-squared	0.0355	0.294	0.314	0.313	0.295

*Notes*: the Table presents OLS regression estimates on the endnodes of the game. The unit of observation is one game—i.e., a pair of matched subjects in one specific repetition of the game. Repetitions are treated as a continuous variable. Variable ‘trust’ is a dummy taking value 1 if a subject agrees, or completely agrees, with the statement: “In general, one can trust other people”. ‘Risk’ represents a self-reported measure, on a 1 to 10 scale, on one’s propensity to take risks (higher values = lower risk aversion), and is treated as a continuous variable. ‘Complexity’ is a measure, on a 1 to 5 scale, of one’s perception of the complexity of the task (higher values = higher complexity), and is treated as a continuous variable. Variable ‘Errors’ is the number of mistakes recorded before completing the control quiz. ‘Timequiz’ is the number of seconds taken before correctly completing the control quiz. ‘min()’ indicates that the minimal value in the pair of matched subjects is considered. Standard errors are clustered at the session level. P-values:

*: <0.1;

**: <0.05;

***: <0.01;

Furthermore, to justify our choice, we gathered evidence on contagion effects within sessions in the data. We perform an analysis of variance for each treatment, to assess how the variance across sessions evolves over time, relative to the variance within each session. Considering the three initial repetitions, we reject the null that sessions come from populations with the same variance for treatments *Formula* and *Decomposed* (Bartlett’s test, significance level: .05). We cannot reject the same null for any treatment in the last three repetitions. This suggests that variance within each session does not decrease more rapidly than variance across sessions (overall, the variance between sessions accounts for between 1 and 4 percent of the overall variance within each treatment). Similarly, within-treatment regressions cannot detect different time trends across sessions, or any significant variation in session effects in latter relative to earlier periods (all tables are available upon request).

**Fig 4:**
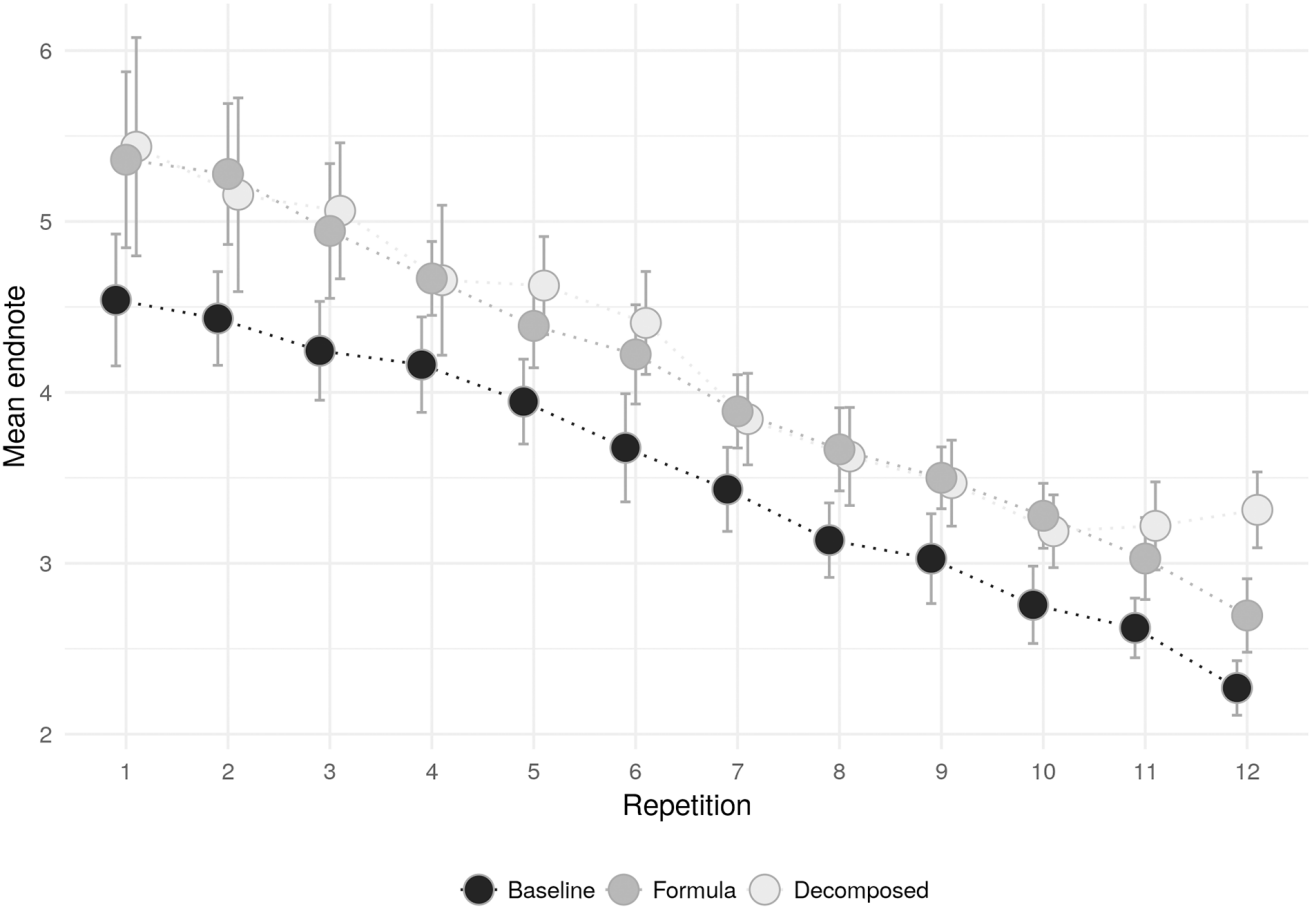
Mean endnode by treatment and repetition. Notes: the Figure shows, separately for each treatment, the evolution of the average endnode over the twelve repetitions of the game. Each dot represent the average endnode for one repetition, and is plotted together with its 95% confidence interval.

Fig 4 shows how the average endnode evolved over repetitions. We observe gradual learning toward equilibrium in each treatment. Conditions *Formula* and *Decomposed* overlap in almost all repetitions. The difference of these two with the baseline appears to be stable. Column (2) in Table 2 confirms that the linear learning trend is not significantly different across treatments: in all conditions, we detect an average significant shift in the direction of equilibrium of about $\frac{1}{5}$ of an endnode for each repetition.

Result: *In conditions Formula and Decomposed, subjects end the game significantly later than in condition Tree*. *These differences are stable over rounds*. *There are no significant differences between conditions Formula and Decomposed*.

In column (3) in Table 2 we add a set of controls. Controls are measures of risk preferences, perceived complexity and trust coming from the final questionnaire, as well as the time employed to complete the control quiz and the number of errors recorded before completing it correctly.

Since our unit of observation is one pair of matched subjects, we need to transform individual variables into pair-level ones. We do so by attaching to each matched pair the minimal value of the two individual ones. That is, the highest level of risk aversion in the pair (which is the lowest value of the response to the SOEP question); the lowest level of perceived complexity; the lowest level of trust; the shortest time to complete the questionnaire and the least number of errors. The rationale for this choice is as follows. More risk averse subjects should end the game earlier for any subjective probability that the other chooses ‘stop’ at the following node. People who have a better understanding of the game may also choose ‘stop’ earlier, *ceteris paribus*. Finally, more trusting subjects may expect that their opponent is more likely to pass at the following node. Data confirm that subjects choosing ‘stop’ have significantly higher risk aversion and are significantly less trusting than their opponent. The statistics for complexity and quiz measures are in line with the above-mentioned hypotheses, although differences are not significant. Results are nonetheless robust to taking the mean pair value or the value of the subject stopping the game.

We include measures of perceived complexity and related to the control quiz in order to check that treatment effects are not due to lack of understanding of the instructions of the game, but, rather, to hampered strategic reasoning when playing the game. In principle, one may think that self-reported measures of perceived complexity should capture strategic complexity faced by subjects and, in particular, differences across treatments. We do not believe the measure is well suited for that. Our manipulations alter the strategic complexity marginally. It is unlikely that subjects can perceive that clearly, and even less likely that these perceptions can be captured by a categorical variable on a one to five scale, with no common benchmark of what, e.g., a complexity of three means. Qualitatively, subjects find the task more complex in *Formula* and *Decomposed*, where they also take more time and make more mistakes before clearing the control questions (in particular, all pairwise differences with *Decomposed* are significant). Our estimates of the treatment effects are, indeed, robust to the inclusion these controls.

Finally, we check whether risk aversion and trust interact differently with different treatments. Risk aversion may have a stronger effect in condition *Decomposed*, because the potential loss implicit in passing is made explicit. Low trust could also have a stronger effect in condition *Decomposed*, because stage payoffs frame the centipede game as a repeated trust game. We test for the presence of differentiated effects of risk and trust attitudes, using interaction terms in the regressions in column (4) and (5) of Table 2. We find no significant difference in the effects of these variables across treatments, and the estimation of our treatment effects is robust to the inclusion of these interaction terms. This implies that treatment differences are not mediated by preferences, according to this analysis.

## Discussion and conclusion

According to our interpretation of the results, a simple reduction in the accessibility of information in our centipede game shifts endnodes further away from the equilibrium, with no sign of convergence with respect to the baseline treatment through repetitions.

[25] report on a manipulation that is partly similar to ours. Subjects play a centipede game with incomplete information, where the game moves automatically to the next decision node, unless the player presses ‘stop’ within ten seconds. The player that ends the game gets an amount that increases over decision nodes, the other gets nothing. In one treatment, subjects are shown the extensive-form game. In another, they are explained the rule that determines payoffs, framed as a sequential Dutch auction. Subjects have private fixed values for an object. They have sequential opportunities to buy it, and the price of the object decreases of a constant amount in every decision node.

This manipulation shares many features with our *Formula* condition. [25] find subjects take earlier under it, while we find the opposite in *Formula*. Many features of the game and the design distinguish our experiment from theirs—i.e., we do not perform a replication of [25]—and we cannot identify the reason behind the different results. It is nevertheless useful to briefly discuss the most prominent features that distinguish our studies and are arguably responsible for the different results.

First, [25] use an incomplete information game. While this is strategically identical to a centipede game under any belief about the opponent’s payoffs, it adds one layer of complexity and uncertainty to the baseline game. Second, payoffs of the game are different. The player who does not end the game always earns zero. The payoff for the player who ends the game, instead, is relatively flat over the course of the game. Thus, the environment is much more competitive than our centipede game, strict efficiency gains are not possible, and the incentives to push the game forward are reduced. Third, decisions are taken under time pressure. As shown in [5], subjects switch to simpler heuristics, act less strategically, and overlook the opponents’ payoffs when deciding under time pressure. Finally, the manipulation in [25] also includes a language shift: in the ‘auction’ version, in order to take the player must “Acquire” a good at a certain “Price”. This type of market frame may elicit more competitive or selfish attitudes [26, 27].

We argue that in a more complex and competitive game with time pressure, the joint effect of a marginal increase in complexity—having to compute future payoffs—and of the switch to a market frame may push subjects to ‘take the money and run’, disregarding all information about future nodes. This would shift the distribution of endnodes leftward. In a standard centipede game, instead, a marginal increase in complexity throws sand in the gearbox of strategic thinking—for instance, reducing the depth of backward reasoning or making errors more likely—and this would shift the distribution of endnodes rightward. We find evidence consistent with this latter interpretation both in the *Formula* and in the *Decomposed* condition. This supports the view that bounded strategic reasoning is a central determinant of departures from equilibrium in the centipede game.

On a more general note, putting into perspective our results and those in [25] shows once more that behavior can be extremely sensitive to subtle details of the presentation of a game. In particular, behavior may react in a non-monotonic and discontinuous way to marginal increases in complexity. As already noted in [3], such an increase may marginally hamper strategic thinking (as in our case), or trigger a shift to a simpler heuristic, depending on the baseline strategic environment.

## Appendix

In the following, the English instructions for condition “Tree” are reported. In brackets are detailed the changes made to adapt the instructions to condition “Formula” (F) and “Decomposed” (D). The original German instructions are available upon request.

### Introduction: Common to all conditions

Welcome and thanks for your participation to this experiment. Please remain silent and switch off your mobile phone. Please do not talk and raise your hand if there are any specific questions during the experiment: an experimenter will come to your place and answer your concerns individually. If you violate these rules, we will have to exclude you from the experiment and all payments.

You receive a 2.5 euro show-up fee for taking part in the experiment. Please read the following instructions carefully. Prior to the experiment, you will have to answer a few questions testing your comprehension of these instructions. Please note that, for convenience, the instructions are written in male gender, but refer to both genders equally.

During the experiment you are going to use ECU (Experimental Currency Units). At the end of the experiment, earned ECU will be converted into euros at an exchange rate of

1euro=1ECUs.

You will take part in a game played by two persons, white and black. You will be randomly assigned the role of white or black, which you will keep for the whole experiment.

The game consists of 12 ordered decision rounds (first round: round = 1, …, last round: round = 12). The players play sequentially. When it is his turn to play, each player can choose between STOP and CONTINUE.

If a player chooses STOP, the game ends.

If a player chooses CONTINUE, the game continues, and the other player faces a choice between STOP and CONTINUE.

White plays first; if he chooses STOP, the game ends, but if he chooses CONTINUE, black is called to play and decide whether to STOP or CONTINUE, and so on. Thus each player has at most six choices, with white choosing at round 1, 3, 5, 7, 9, and 11 and black choosing at round 2, 4, 6, 8, 10, and 12. The sequence of choices continues until one player chooses STOP. If both players choose CONTINUE in every decision round the game ends at round = 13.

### Payoff information: Different across conditions

#### Tree

Below you can see a representation of the game. The game starts from the utmost left. The color of the circles identifies which player has to decide; the numbers in the circle represent the decision round; the numbers in the brackets represent the final payment, in ECU, obtained by each action. In white you see the payoff of white, in black the payoff of black.

[The image shown to the subjects is reproduced above in Fig 1].

#### Formula

When a player chooses STOP at round = r, the value for him is 4 times the current round, that is:

$$V_{STOP}=4\cdot r$$

The value for the other player is 1 times the current round, that is

$$V_{OTHER}=1\cdot r$$

#### Decomposed

Below you can see a representation of the game. The game starts from the utmost left. The color of the circles identifies which player has to decide; the numbers in the circle represent the decision round; the numbers in the brackets represent the change in payments, in ECU, on top of what you have already earned, resulting from each action. The amount you have earned so far will always be visible on your screen. In white you see the payoff of white, in black the payoff of black.

[The image shown to the subjects is reproduced above in Fig 2].

### Actual play of the game and payment (differences in brackets)

When it is your turn to play, you will see a screen that:

1. reminds you of the current round of the game,
2. shows you the amount you and your partner earn if you choose STOP and
3. asks you to choose between STOP and CONTINUE.

You have 30 seconds to reach a decision. You can revise your choice at any time within the 30 seconds. The choice is final when you press OK.

When it is not your turn to play, you will see a screen that:

1. reminds you of the current round of the game and
2. shows you the amount you and your partner earn if your partner chooses STOP

Your partner has 30 seconds to make a decision as well. The game continues until one player chooses STOP or if the last decision round {*Tree*, *Decomposed*: on the right of the above representation} is reached.

{*Tree*: When the game finishes, payoffs are assigned according to the values in the picture above. You will be paid according to the values that appear at the point in which the game stops}.

{*Formula*: When the game finishes, payoffs are assigned according to the formula detailed above. You will be paid according to the decision round in which the game stops}.

{*Decomposed*: You start with a payoff of 4 if you are white, 1 if you are black. After each decision, your earnings will be updated according to the values that appear in the picture above. You will be paid what you have earned up to the point at which the game stops}.

You will play the game 12 times. Each time, you will form a couple with a new player chosen at random from the other participants in this room. You will never play the same partner twice. Your partners will never play one another.

Only one game of the 12 you play will be paid. At the end of the experiment, one number between 1 and 12 will be selected at random by the computer, and the corresponding game will be paid.

For the chosen game the result of you and your partner’s action will be shown on the screen, and your final payoff will be computed.

Should you have any questions, please raise your hand now. An experimenter will come to your place and answer your questions in private.
